# Use of Croton Chloral Hydrate in Photophobia

**Published:** 1876-09

**Authors:** P. Owen-Jones


### THE USE OF CROTON CHLORAL HYDRATE IN PHOTOPHOBIA.

By P. OWEN-JONES, M.D.

(Read before the Chicago Medical Society, Juue 19, 1876.)

[Dr. Owen-Jones remarked in beginning that the cases here reported were treated in Guy's Hospital, London, under the direction of Mr. Bader.—Ed.]

(1) Ann B., aged 18, suffering from syphilitic corneo-iritis of both eyes with photophobia, commenced to take grs. v. doses of croton chloral hydrate three times a day, on the 24th Dec., 1873. A solution of daturin (gr. j. to ℥j. of water) one drop to be dropped into both eyes every ten minutes for an hour each evening. Eight days after the photophobia had greatly diminished, and on the twenty-third day, Jan. 16th, 1874, she was discharged cured.

(2) Mary C., aged 11, suffering from pannus with granulations and photophobia, commenced to take grs. v. of croton chloral hydrate three times a day, on Jan. 16th, 1874. A solution of daturin (gr. j.—℥j. of water), one drop twelve times in an hour each evening. On the seventh day there was no improvement, and the dose was increased to grs. x. three times a day. On the fifteenth day there was a very slight improvement in left eye only. The dose was increased to grs. xij. On the twenty-sixth day the drops were omitted and the dose increased to grs. xv. On the twenty-eighth day, there being no improvement, the medicine was omitted and the lids were touched with greenstone seven or eight times a day.

(3) Mary S., aged 19, suffering from syphilitic corneo-iritis with great photophobia, commenced to take grs. x. doses of croton chloral hydrate three times a day, on Dec. 24th, 1873. A solution of atropine (grs. ij.—℥j. of water) twelve drops in one hour each evening. On the thirteenth day the photophobia had almost subsided. On the nineteenth day the photophobia had entirely gone.

(4) Florence B., aged 8, suffering from syphilitic corneo-iritis with epiphora and photophobia, commenced with grs. v. doses three times a day, on Jan. 13th, 1874. A solution of atropine (gr. j.—℥j. of water) six times in an hour each evening. On the the fourth day there was a very marked improvement, which continued up to the twenty-fourth day, when a dense fog caused a return of the photophobia. On the thirty-second, patient was discharged cured.

(5) Celia S., aged 24, suffering from closed pupil, ciliary redness and great photophobia, trusion—1. Commenced with grs. x. doses three times a day, Jan. 13th, 1874. A solution of atropine (grs. iv.—℥j. of water) to be used morning and evening. The left eye was worse than right. On the fourth day there was a slight improvement in photophobia. On the tenth day patient was able to look at the light with only slight discomfort. On the fourteenth day there was no photophobia in right eye. On the eighteenth day the photophobia had ceased in both eyes.

(6) Mary D., aged 15, suffering from pannus, with granulations and photophobia, commenced to take grs. x. doses three times a day, a solution of atropine (grs. ij.—℥j.) to be used three times a day, Jan. 21st, 1874. On the second day there was a slight improvement. Drops omitted. On the nineteenth day there was a great improvement in photophobia. Dose increased to grs. x. four times a day. On the twenty-first day the dose was increased to grs. xv. four times a day. On the twenty-seventh day the photophobia had ceased and the medicine was omitted. The lids were ordered to be touched with greenstone three times a day.

(7) Ellen B., aged 7, suffering from corneitis with excessive photophobia, commenced to take grs. viij. doses three times a day, Feb. 5th, 1874. On the fourth day there was a marked improvement in photophobia. On the eighth day the photophobia suddenly returned as bad as at first. The medicine was continued for several days, but without producing any good result.

(8) Clara S., aged 15, suffering from corneo-iritis with excessive photophobia, so that it was necessary to keep her entirely in the dark, commenced to take grs. x. four times a day, Feb. 10th, 1874. On the second day there was already a slight improvement. On the third day patient complained of a bad headache. On the sixth day the pain in head was better. Medicine was reduced to grs. v. There was a gradual improvement up to the twentieth day, when patient left the hospital.

(9) Mary B., aged 49, suffering from closed pupil with internal neuralgia of forehead, commenced to take grs. xv. doses three times a day. On the fourth day the dose was increased to grs. xx., but without producing any marked alleviation from pain.

If now we look at the results of these cases, we see that Nos. 1, 3, 4, 5 and 8 were markedly benefitted by the drug, and No. 6 to a great extent. These cases, Nos. 1, 3, 4, 5 and 8, were the subjects of well marked congenital syphilis. All the cases were the worst I could pick out from among the out patients attending at the hospital. It is right perhaps to state that most of these patients came from miserable dirty homes, where they were probably only half fed; so that the good food and nursing of the hospital may have had something to do with the result.

As regards the dose of the medicine, it will be seen that various quantities were given, the smallest being grs. v. three times a day, and the largest grs. xx., without producing, as far as I could detect, any unpleasant symptom. The only complaint was the exceedingly nasty taste of the drug, which was given combined with syrup of lemons, about ʒ iij. to ℥ j. of water, in which it was found to be more soluble than in water alone. In only one patient, No. 6, did it cause any feeling of sleepiness. Dr. Bader concluded, from the result of these cases, that it was only in diseases of the eye which were due to syphilis we could expect any marked and permanent benefit.

